# What Is the Effect of Basic Emotions on Directed Forgetting? Investigating the Role of Basic Emotions in Memory

**DOI:** 10.3389/fnhum.2016.00378

**Published:** 2016-08-08

**Authors:** Artur Marchewka, Marek Wypych, Jarosław M. Michałowski, Marcin Sińczuk, Małgorzata Wordecha, Katarzyna Jednoróg, Anna Nowicka

**Affiliations:** ^1^Laboratory of Brain Imaging, Neurobiology Centre, Nencki Institute of Experimental Biology – Polish Academy of SciencesWarsaw, Poland; ^2^Faculty of Psychology, Warsaw UniversityWarsaw, Poland; ^3^Laboratory of Psychophysiology, Department of Neurophysiology, Nencki Institute of Experimental Biology – Polish Academy of SciencesWarsaw, Poland

**Keywords:** directed forgetting, discrete emotions, disgust, Nencki Affective Pictures System

## Abstract

Studies presenting memory-facilitating effect of emotions typically focused on affective dimensions of arousal and valence. Little is known, however, about the extent to which stimulus-driven basic emotions could have distinct effects on memory. In the present paper we sought to examine the modulatory effect of disgust, fear, and sadness on intentional remembering and forgetting using widely used item-method directed forgetting (DF) paradigm. Eighteen women underwent fMRI scanning during encoding phase in which they were asked either to remember (R) or to forget (F) pictures. In the test phase all previously used stimuli were re-presented together with the same number of new pictures and participants had to categorize them as old or new, irrespective of the F/R instruction. On the behavioral level we found a typical DF effect, i.e., higher recognition rates for to-be-remembered (TBR) items than to-be-forgotten (TBF) ones for both neutral and emotional categories. Emotional stimuli had higher recognition rate than neutral ones, while among emotional those eliciting disgust produced highest recognition, but at the same time induced more false alarms. Therefore, when false alarm corrected recognition was examined the DF effect was equally strong irrespective of emotion. Additionally, even though subjects rated disgusting pictures as more arousing and negative than other picture categories, logistic regression on the item level showed that the effect of disgust on recognition memory was stronger than the effect of arousal or valence. On the neural level, ROI analyses (with valence and arousal covariates) revealed that correctly recognized disgusting stimuli evoked the highest activity in the left amygdala compared to all other categories. This structure was also more activated for remembered vs. forgotten stimuli, but only in case of disgust or fear eliciting pictures. Our findings, despite several limitations, suggest that disgust have a special salience in memory relative to other negative emotions, which cannot be put down to differences in arousal or valence. The current results thereby support the suggestion that a purely dimensional model of emotional influences on cognition might not be adequate to account for observed effects.

## Introduction

Beneficial impact of emotion on memory is well-documented. Emotionally arousing stimuli are better remembered than emotionally neutral ones (Rubin and Friendly, 1986; Bradley et al., 1992; Palomba et al., 1997; Ochsner, 2000) and memories of emotional events have a persistence and vividness that other memories seem to lack (Christianson, 1992). Memory advantage has been shown to be particularly strong for negative when compared to neutral stimuli (e.g., Danion et al., 1995; Phelps et al., 1997; Michalowski et al., 2014; Riegel et al., 2016a). In addition, forgetting of negative stimuli is relatively more difficult than neutral stimuli (Otani et al., 2012; Brandt et al., 2013).

The studies of emotional memory typically focused on the memory-enhancing effects of emotional dimensions of arousal and valence. However, little is known about the extent to which basic emotions evoked by the emotional stimuli could have distinct effects on memory over and above their dimensional influence. Although dimensional accounts of emotion are informative, the influence of discrete emotions should not be underestimated (Levine and Pizarro, 2006). Defined by Ekman (1999), basic emotions (happiness, surprise, sadness, fear, anger, and disgust) can be considered as psychophysiological entities that are behaviorally observed and cross-culturally distinguished. Different basic emotions are associated with characteristic patterns of cognitive appraisal (Scherer, 1987), action readiness (Frijda, 1987), and risk perception (Lerner and Keltner, 2001). There is still a debate on whether or not they depend on distinct neural substrates (Kober et al., 2008; Lindquist et al., 2012). Even though many studies showed an association between specific brain structures and particular basic emotions, there is little consensus whether basic emotions are linked with both consistent and discriminable regional brain activations (Vytal and Hamann, 2010). It seems that more complex, network-based representations of emotion are needed rather than simple one-to-one mappings between emotions and brain regions (Saarimäki et al., 2015; for review see Kragel and LaBar, 2014).

From a wide range of negative emotions, fear and recently disgust have gained the most attention in cognitive studies. Both emotions are highly arousing and negatively valenced and they are associated with a strong motivation to avoid a particular object or situation (Woody and Teachman, 2006; Krusemark and Li, 2011). However, they are different at the level of physiological responses (Susskind et al., 2008). Fear is a response to danger that is associated with a cascade of somatic and autonomic adjustments including increases in heart rate, skin conductance and respiration (Fredrikson, 1981; LeDoux, 2003) as well as – depending upon the distance of danger – freezing or escape behaviors (Fanselow, 1994; Löw et al., 2015). Disgust is a powerful affective feeling that can protect us from illness or intense feelings of revulsion, nausea and possible death. Other than fear, disgust stimuli produce reduced blood pressure, heart rate deceleration, and decreased respiration rate (Critchley et al., 2005; Ritz et al., 2005; Stark et al., 2005). Additionally, while the fear is believed to be largely automatic, the disgust presumably develops more slowly and depends more on focal attention (Santos et al., 2008). Consequently, it has been shown that attention disengagement is more difficult from disgust-related than from fear-related stimuli (Cisler et al., 2009; van Hooff et al., 2013, 2014). There is also evidence for a better memory recall in the case of disgusting over frightening stimuli for words (Charash and McKay, 2002) and images (Croucher et al., 2011). Disgust enhancement of memory recall remained significant even when attention at encoding, arousal, visual salience, and conceptual distinctiveness were controlled (Chapman et al., 2012). Fear and disgust can be contrasted with sadness that is associated with lack of energy, inertia, loss, despair, helplessness, disappointment, and sorrow. Stimuli eliciting sadness result in a reduced heart rate, skin conductance level, and respiration frequency when compared to fear-evoking (Kreibig et al., 2007) as well as a lower skin conductance level than disgust stimuli (Lang et al., 1993). Sadness is associated with an internal attention focus and a reduction in general alertness (Finucane et al., 2010). Much less is known about the cognitive specificity of sadness in relation to other basic emotions.

Together, if fear, disgust, and sadness indeed affect attention and memory differently then it seems unjustified to treat them as one single category of negative emotions. As a result of such approach the effects of different basic emotions may cancel out leading to a substantial attenuation of the observed effects. Therefore, in the present paper we sought to examine the modulatory effect of basic emotions on memory processes both on behavioral and neural levels. Specifically, we were interested whether remembering as well as forgetting of complex pictorial stimuli would be similar or rather dissimilar in the case of photographs eliciting different basic emotions. In order to achieve these goals we applied the item-method directed forgetting (DF) paradigm (MacLeod, 1998, 1999).

Briefly, in the item-method DF paradigm, study items are individually cued to-be-remembered (TBR) or to-be-forgotten (TBF) on a trial-by-trial basis: ‘remember’ (R) or ‘forget’ (F) instruction follows the presentation of each study item. Afterward, memory is tested for all items, irrespective of the previous memory instructions (MacLeod, 1998, 1999). Recall and recognition of TBF items is generally impaired compared with TBR items. This effect is known as the DF effect (Bjork, 1989; Johnson, 1994; Basden and Basden, 1996; MacLeod, 1998, 1999).

Item-method DF effect may be explained by the selective encoding hypothesis (Bjork, 1972) suggesting that each item is maintained in active memory until the cue is presented and then, if the cue is to remember the item (TBR), it is processed further (i.e., rehearsed). In contrast, when the cue is to forget (TBF), than that item is dropped from active memory and it is not further rehearsed. As a consequence, only TBR are selectively encoded and therefore better remembered than TBF. However, a growing body of evidence indicates that active inhibitory processing is triggered by the F cue in the item-method DF paradigm (Wylie et al., 2008; Fawcett and Taylor, 2010; Lee and Hsu, 2013), resulting in successful forgetting of TBF items.

DF effects were reported not only for verbal but also for non-verbal (pictorial) stimuli (Lehman et al., 2001; Hourihan and Taylor, 2006; Hauswald and Kissler, 2008; Hourihan et al., 2009; Quinlan et al., 2010; Nowicka et al., 2011; Zwissler et al., 2011, 2015). In the context of the current study it is of a great interest that DF effects were observed for both neutral and emotionally negative images and they were stronger for the former and weaker for the latter (Nowicka et al., 2011). In the case of emotionally positive images, DF was attenuated in reference to DF for neutral pictures (Zwissler et al., 2011).

Only a limited number of fMRI studies examined neural underpinnings of flexible memory control in the case of emotionally negative images (Depue et al., 2006; Nowicka et al., 2011). Using DF paradigm, Nowicka et al. (2011) reported that intention to forget and successful forgetting of emotionally negative images were associated with widespread activations in the frontal, parietal and occipital areas, whereas in the case of neutral images, they were associated with just one cluster of activation in the right lingual gyrus (Nowicka et al., 2011). The other fMRI study (Depue et al., 2006) that investigated the issue of forgetting of emotional pictorial material used a different than DF paradigm of flexible memory control, i.e., the think/no-think paradigm (Anderson and Green, 2001; Anderson, 2003; Anderson et al., 2004; Anderson and Levy, 2009). Suppression of retrieval of aversive scenes in ‘no-think’ trials was associated with reduced activation in the amygdala, a structure implicated in emotion processing. Importantly, during no-think trials, both the hippocampus and amygdala were not simply less engaged than they were during ‘think’ trials, they were also less active than they were when people simply looked passively at an empty screen, suggesting that overcoming retrieval involves actively disengaging these brain regions (Depue et al., 2006).

In the present study, fMRI data were analyzed using both the whole-brain and the region of interest (ROI) approach. Since both human and animal studies suggest that preferential memory for emotional events depends on the influence of the amygdala upon the hippocampus (McGaugh, 2004), our analyses focused on these two ROIs. Additional ROI was defined in the right superior and middle frontal gyrus (BA9/10), as this area was consistently more active during intentional compared to incidental forgetting (Anderson and Hanslmayr, 2014).

## Materials and Methods

### Subjects

Twenty-two healthy right-handed women participated in the study. All subjects gave their written informed consent to the study. They were mostly students from University of Warsaw. The data from four participants were excluded from analyses due to technical problems. Data from remaining 18 subjects (mean age = 22.02 years, *SD* = 0.96) were analyzed. The local Research Ethics Committee at Faculty of Psychology University of Warsaw approved the experimental protocol of the study.

### Stimuli

The set of stimuli consisted of images taken from the Nencki Affective Picture System (NAPS, Marchewka et al., 2014b), the International Affective Picture System (Lang et al., 2005) and Flickr, which is an image and video hosting website. For Flickr images only those under the Creativity Commons license were used^1^

**FIGURE 1:**
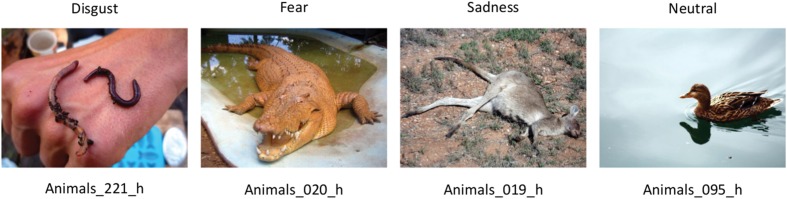
**Exemplary stimuli used in the current study eliciting disgust, fear, and sadness together with a neutral item.** Note that the exemplary stimuli are only from Animals category and are not extreme for each emotion. Numbers represent order of the stimuli in the NAPS database.

The selection of NAPS images eliciting disgust, fear, or sadness was based on basic emotions ratings (Riegel et al., 2016b). Stimuli from Flickr and IAPS were chosen in order to match the number of stimuli in each emotion category and semantic content. Disgust eliciting stimuli contained realistic pictures of deformed or rotten body parts, vomiting people, diseased or rotten animals, insects and warms, rotten food, meat, dirty toilets, excrements and garbage. Sadness eliciting stimuli contained pictures of crying people, sick, elderly, homeless, starving or wounded people, starving or sick animals, collapsing buildings, coffins and graveyards. Fear eliciting stimuli contained images of snakes, spiders, sharks, wolves, natural disasters, assault, war and riots, drowning people, prison, guns, scalpels, etc. Neutral stimuli contained images of people in neutral situations not expressing emotions, birds and farm animals, vegetables and mushrooms, buildings and vehicles. Exemplary stimuli are depicted in **Figure 1.**

The NAPS database is freely available to the scientific community for non-commercial use by request at http://naps.nencki.gov.pl. The set of stimuli used in the current study can be obtained from the corresponding author.

### Procedure

Stimuli were fully counterbalanced in respect of their content and basic emotional categories between the study and test phases and between to-be-remembered (TBR) and to-be-forgotten (TBF) conditions. They were displayed using Presentation software (Neurobehavioral Systems, Inc., Albany, CA, USA) on MR compatible high-resolution LCD monitor positioned at the back of MR scanner. Subjects were able to see the stimuli through the mirror system placed in the MR coil. The study consisted of three parts – study phase (in MR scanner), test phase and subjective ratings of valence, arousal, and intensities of basic emotions.

In the study phase, 240 pictures (120 neutral and 120 emotionally negative: 40 pictures representing each negative basic emotion) were displayed; half were followed by the instruction to-be-remembered (TBR) and the other half by the instruction to-be-forgotten (TBF). The order of experimental trials was pseudorandom with the constraint of no more than three consecutive trials with the same instruction type or the same picture category. The images were displayed for 1 s each, followed by the fixation cross presented for 1 s and then the memory instruction [i.e., the word REMEMBER (R) or FORGET (F)] for 1.5 s. The post cue interval varied pseudorandomly from 5 to 7 s.

After around 30-min-long break subjects underwent the test phase outside the MR scanner. All the previously used stimuli were re-presented together with 240 new pictures (120 neutral and 120 emotionally negative: 40 pictures for each negative basic emotion). Subjects had to categorize each picture, irrespective of the F/R instruction, as old (displayed in the study phase) or new, using a standard response pad. Trials were mixed pseudorandomly and fully counterbalanced with respect to all experimental conditions (old/new and emotionally negative/neutral stimuli). All images were presented for 2 s, with the inter stimulus interval of 5 s.

Finally, after an additional 30-min break (subjects were able to go outside the lab or/and have a soft or hot drink) participants were asked to rate previously seen 480 images. Each stimulus was presented for 2 s after which a rating panel was presented with a small version of the pictures above. Subjects were asked to rate on five different scales how they felt when viewing each picture. The first three scales described the intensity of the emotion (disgust, sadness, and fear) elicited by the picture, going from 1 = “none” to 5 = “a lot.” Participants also rated valence and arousal using a nine-point Self Assessment Manikin Scale (SAM; Bradley and Lang, 1994) going from 1 = “negative” (valence) and “calm” (arousal) to 9 = “positive” (valence) and “aroused” (arousal).

### MRI Data Acquisition

Magnetic resonance imaging was carried out using a 3-Tesla Trio MRI scanner (Siemens Medical Solutions) equipped with a 32-channel phased array coil. A high-resolution T1-weighted image (T1w) was acquired using the following acquisition parameters: TR: 2530 ms, TE: 3.32 ms, flip angle: 7°, 176 slices with an in-plane resolution of 1 mm^3^, field of view: 256 mm, slice thickness: 1 mm. Functional images were acquired using an echo planar imaging pulse sequence (field of view: 224 mm, matrix: 64 × 64, slice thickness: 3.5 mm, TE: 25 ms, TR: 2000 ms, flip angle: 90°). Thirty-four contiguous, oblique-axial images oriented parallel to the anterior–posterior commissural plane were acquired with a total of 1150 volumes.

### fMRI Data Analysis

Behavioral records were used to sort the fMRI data based on the memory instruction and behavioral outcome. Imaging data were pre-processed and analyzed using Statistical Parametric Mapping software (SPM12, Wellcome Department of Cognitive Neurology). First, functional images were motion corrected. Then, the structural images from single subjects were co-registered to the mean functional images. The unified segmentation (Ashburner and Friston, 2005) was used to separate anatomical images into gray matter, white matter and other tissues. High-dimensional Diffeomorphic Anatomical Registration Through Exponentiated Lie Algebra (DARTEL, Ashburner, 2007) was used to create a study-specific template (Marchewka et al., 2014a), which was then affine registered with Montreal Neurological Institute (MNI) space. The functional images were normalized using compositions of flow fields and template affine transformation parameters. Finally, the normalized functional images were smoothed with an 8 mm full-width at half-maximum isotropic Gaussian kernel.

Whole brain statistical fMRI analyses were performed first at the subject level and then at the group level. At the subject level timings for all experimental conditions (to-be-remembered and remembered – TBR_R, to-be-remembered but forgotten – TBR_F, to-be-forgotten and forgotten – TBF_F, to be forgotten but remembered – TBF_R) for each type of emotions (disgust, fear, sadness) and neutral stimuli were entered into the design matrix as well as head movement parameters. The hemodynamic response was modeled using canonical hemodynamic response function (HRF) implemented in SPM software.

The number of trials with images evoking basic emotions in each of the conditions (TBR_R, TBR_F, TBF_R, and TBF_F) was too low to include this factor in the whole brain analyses at the group level. Therefore, we collapsed trials across the emotion conditions in order to examine the effects of intentional remembering and forgetting in general. Intentional remembering was examined by comparing intentionally remembered trials (TBR_R) with incidentally remembered ones (TBF_R) and intentional forgetting by comparing intentionally forgotten trials (TBF_F) trials with incidentally forgotten ones (TBR_F). On a group level a voxel-wise height threshold of *p* < 0.001 (uncorrected) combined with a cluster-level extent threshold of *p* < 0.05 (corrected for multiple comparisons using the family wise error rate) was employed for whole brain analyses. Coordinates of significant effects are reported in MNI space. XjView was used to identify the activated structures^2^

The effect of basic emotions on intentional remembering and forgetting was more closely explored in the ROIs analysis. Similar as above, 16 conditions (TBR_R, TBR_F, TBF_R, TBF_F) × (disgust, fear, sadness, and neutral) and six head movements parameters were entered into the design matrices. However, since the ratings of arousal and valence differed significantly between basic emotional categories two additional vectors representing arousal and valence ratings were entered into design matrix as covariates of no interest. For each stimulus arousal and valence ratings were averaged across subjects. Then the ratings were time-aligned with the corresponding stimuli onsets and durations and convolved with HRF resulting in the covariate vectors. The additional vectors were put as the last columns of the design matrix, however, manual permutation of the columns before model estimation and putting the regressors as the first columns did not change the results. Five anatomical areas created using WFU_pickatlas verision 3.0.5^3^ (Lancaster et al., 2000; Maldjian et al., 2003) were included: left and right amygdala, left and right hippocampus and right superior and middle frontal gyrus. For these five ROIs, contrast estimates from the model including arousal and valence as covariates were extracted for each condition using Marsbar toolbox (see **Figure 6**). Repeated-measures ANOVAs (with Greenhouse–Geisser corrections for non-sphericity) were used with factors: instruction (TBR and TBF), memory (remembered or forgotten) and emotion (neutral, disgust, fear, and sadness).

## Results

### Behavioral Results

#### Recognition Rates for TBR_R, TBF_R Trials and False Alarms

The effectiveness of the DF paradigm was tested firstly in line with previous studies (Wylie et al., 2008; Nowicka et al., 2011; Lee and Hsu, 2013) by analyzing recognition rates for TBR_R and TBF_R trials as well as false alarms (new stimuli judged as old ones) using repeated measures ANOVAs with type of stimulus (TBR_R, TBF_R, and false alarms) and type of emotion (disgust, fear, sadness, and neutral) as factors. The average recognition rates are presented in **Table 1.** Significant main effects of type of stimulus [*F*(2,16) = 144.9, *p* < 0.001] and emotion [*F*(3,15) = 25.9, *p* < 0.001] were revealed as well as their interaction [*F*(6,12) = 4.30, *p* = 0.015]. In line with the DF effect, the recognition rate for TBR items was significantly higher than that for TBF items (TBR_R = 68.5% vs. TBF_R = 57.6%, *p* < 0.001). Additionally, the correct recognition rate for TBF was significantly higher than the false alarms’ rate (*p* < 0.001). Interestingly, the DF effect was observed for each emotion type: disgust (TBR_R = 78.9% vs. TBF_R = 65%, *p* = 0.001), fear (TBR_R = 70.3% vs. TBF_R = 55.8%, *p* < 0.001), sadness (TBR_R = 67.9% vs. TBF_R = 60.4%, *p* = 0.051), as well as neutral images (TBR_R = 57% vs. TBF_R = 49.1%, *p* = 0.035). In the case of TBR_R items, all emotionally charged images had higher recognition rate than neutral images (all *p* < 0.001). Furthermore, pictures evoking disgust were significantly better remembered than pictures evoking sadness (*p* = 0.006) and there was a trend in the same direction for images evoking fear (i.e., disgust > fear, *p* = 0.066). In the case of TBF_R items, all emotionally charged images were better remembered than neutral images (disgust – *p* < 0.001, fear – *p* = 0.053, sadness – *p* = 0.016). Again, TBF pictures evoking disgust had higher recognition rate than pictures evoking fear (*p* = 0.027). In the case of false alarms, images evoking disgust and fear produced more false alarms than pictures evoking sadness or neutral pictures (all *p* < 0.001 besides disgust vs. sadness, *p* = 0.006).

**Table 1:** The recognition rates for correctly recognized to-be-remembered (TBR) and to-be-forgotten (TBF) items as well as false alarms for each emotional category.

Emotion	TBR_R (± SD)	TBF_R (± SD)	FA (± SD)
Neutral	57% (12%)	49.1% (15.8%)	8.2% (6.6%)
Disgust	78.9% (12.2%)	65.6% (13%)	17.4% (13.4%)
Fear	70.6% (16.6%)	56.1% (17.2%)	18.3% (10.8%)
Sadness	67.3% (9%)	59.8% (15%)	7.8% (7%)


#### False Alarm Corrected Measures

**FIGURE 2:**
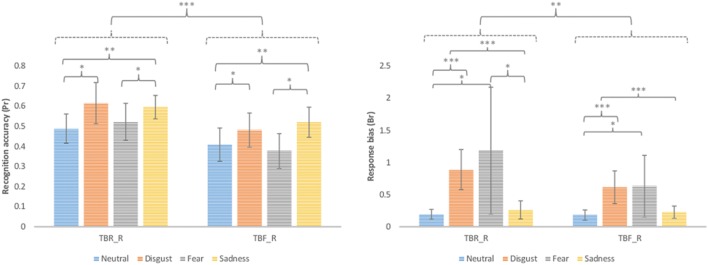
**False alarm corrected recognition accuracy (Pr) and response bias (Br) for correctly recognized TBR and TBF images (TBR_R and TBF_R, respectively).** Error bars represent standard deviation. ^∗^*p* < 0.05; ^∗∗^*p* < 0.01; ^∗∗∗^*p* < 0.001.

**FIGURE 3:**
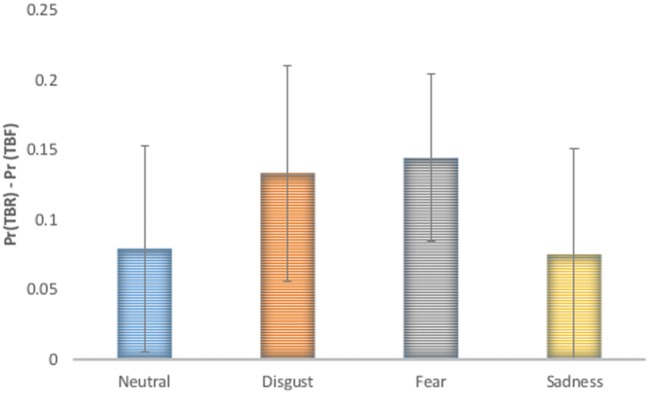
**Directed forgetting effect on false alarm recognition accuracy [Pr (TBR) – Pr(TBF)] was significant for each emotional category and no interaction between basic emotion and stimulus type was revealed**.

Additionally, false alarm corrected measures of recognition accuracy Pr [p (hit)-p (false alarms)] and response bias Br [p (false alarms)/p (1-Pr)] were computed and analyzed using repeated measures ANOVA with type of stimulus (TBR_R, TBF_R) and emotion (disgust, fear, sadness, and neutral) as factors (see **Figure 2**). According to Snodgrass and Corwin (1988) greater Pr values indicate better discrimination between old and new items. Br values higher than 0.5 indicate liberal response criteria (bias to respond “old”) and lower than 0.5 suggest conservative response criteria (bias to respond “new”). In the case of Pr significant effects of type of stimulus [*F*(1,17) = 25.69; *p* < 0.001] and emotion were revealed [*F*(1,15) = 6.95; *p* = 0.001]. TBR_R stimuli had higher Pr values than TBF_R ones. Images inducing disgust and sadness had higher Pr than neutral pictures (*p* = 0.021 and *p* = 0.004, respectively), while fear inducing images had lower Pr than sad ones (*p* = 0.026). DF effects (TBR – TBF) calculated for false alarm corrected recognition accuracy for each emotion is depicted in **Figure 3.** Since the effect was present for all emotions and there was no interaction between emotion type and type of stimulus, it seems that basic emotions have no effect on DF using false alarms corrected recognition.

With respect to Br besides significant effects of type of stimulus [*F*(1,17) = 12.66; *p* = 0.002] and emotion [*F*(1,15) = 4.58; *p* = 0.038], a trend for interaction of these two factors was revealed [*F*(1,15) = 3.10; *p* = 0.088]. Images inducing disgust had higher Br values than sad (*p* = 0.004) and neutral pictures (*p* < 0.001). In the case of disgust or fear inducing images a significant effect of type of stimulus was observed with higher Br for TBR_R than TBF_R items (*p* = 0.023 and *p* = 0.042, respectively). In the case of TBR_R items, disgusting and fear inducing images had higher Br than sad (*p* = 0.001 and *p* = 0.037, respectively) and neutral (*p* < 0.001 and *p* = 0.041, respectively) pictures. In the case of TBF_R items the pattern was largely similar, although the difference between fear and sad images did not reach significance.

In addition we calculated subject-wise Pearson’s correlation of the false-alarm corrected memory performance (calculated as the difference in memory performance for disgust vs. sadness and disgust vs. fear) with differences in arousal and valence ratings (calculated as difference between disgust vs. sadness and disgust vs. fear). In the case of false-alarm corrected memory performance three measures were taken into account: Pr for TBR stimuli, Pr for TBF items and averaged Pr for both to TBR and TBF stimuli. Only the correlation of difference of Prs between disgusting and fearful stimuli for TBF stimuli with difference in arousal between the disgusting and fearful stimuli was on the level of trend, *p* = 0.079, but none of the correlations reached significance (see **Table 2**), which suggests that the observed behavioral effects are rather not confounded by the differences in valence and arousal between emotional categories.

**Table 2:** Subject-wise Pearson’s r correlation of the differences in false-alarm corrected memory performance with differences in arousal and valence for disgust vs. fear inducing images and disgusting vs. sad stimuli.

	Pr_RR (Dis-Fea)	Pr_FR (Dis-Fea)	Pr_total (Dis-Fea)	Pr_RR (Dis-Sad)	Pr_FR (Dis-Sad)	Pr_total (Dis-Sad)
Aro(Dis-Fea)	0.28	0.45	0.40	–	–	–
Val(Dis-Fea)	-0.42	-0.09	-0.29	–	–	–
Aro(Dis-Sad)	–	–	–	-0.03	-0.02	-0.03
Val(Dis-Sad)	–	–	–	-0.26	-0.25	-0.28


*None of the correlations reached statistical significance.*

#### Subjective Ratings

**FIGURE 4:**
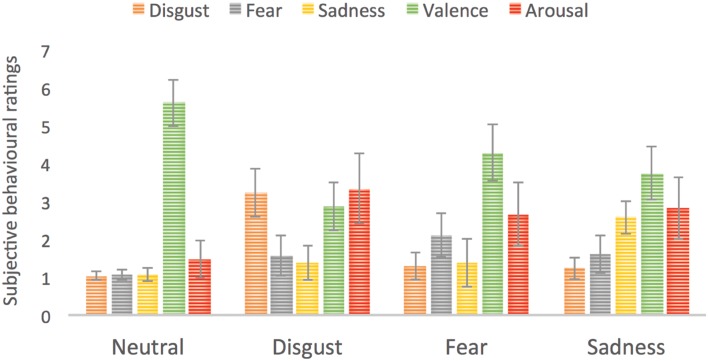
**Subjective ratings for four types of stimuli used in the study.** Error bars represent standard deviation. For the purpose of enhancing visibility of differences we used a scale going from 0 to 7.

Each subject rated all stimuli in the third phase of the experiment. These subjective ratings served as confirmation that presented stimuli successfully evoked specific basic emotions in the group of studied subjects. The average ratings for disgust category on five scales were as following: disgust = 3.2; fear = 1.3; sadness = 1.4; valence = 2.9; arousal = 3.3. The average ratings for fear category were as following: disgust = 1.3; fear = 2.5; sadness = 1.4; valence = 3.1; arousal = 2.7. For sadness inducing stimuli the ratings were: disgust = 1.2; fear = 1.4; sadness = 2.6; valence = 3; arousal = 3.2, whereas for neutral: disgust = 1; fear = 1.1; sadness = 1.1; valence = 5.6; arousal = 1.5. **Figure 4** presents the subjective ratings on five scales reflecting the intensity of each basic emotion as well as valence and arousal. We computed analysis of variance (ANOVA) with basic emotion ratings as well as valence and arousal ratings and picture category (neutral, disgust, fear, or sadness evoking pictures). We found a significant main effect of picture category on arousal ratings [*F*(3,14) = 59.0, *p* < 0.001], where emotionally charged images had higher arousal ratings than neutral ones. Pictures evoking disgust were rated as more arousing than those evoking fear (*p* = 0.005) or sadness (*p* = 0.016). In case of valence ratings again a main effect of picture category was found [*F*(3,14) = 52.5, *p* < 0.001] with lowest valence for disgust, then sadness, then fear followed by neutral images (all *p* < 0.001 besides fear < sadness, *p* = 0.003). Similarly for disgust intensity rating, all stimuli categories differed significantly from each other [*F*(3,14) = 52.5, *p* < 0.001] besides sadness and fear. Pictures from disgust category had the highest disgust rating, followed by pictures from fear and sadness categories and neutral pictures with the lowest rating. In case of fear ratings a significant effect of picture category was found [*F*(3,14) = 14.1, *p* < 0.001] with pictures from fear category having the highest fear rating than all other pictures (*p* < 0.001). Other emotional pictures were rated as more intensive on fear rating than neutral pictures (disgust – *p* = 0.009, sadness – *p* = 0.004). For sadness ratings a main effect of picture category was revealed [*F*(3,14) = 36.5, *p* < 0.001] with pictures from sadness category having the higher sadness ratings than all other picture categories (*p* < 0.001). Again emotionally charged pictures were rated as more sadness evoking than neutral pictures (disgust – *p* = 0.009, sadness – *p* = 0.024).

Since we found significant differences in subjective ratings of valence and arousal between different basic emotional categories, we tried to control for these confounds by performing logistic regression on the item level. In the model we used continuous regressors for valence and arousal, dummy-coded distinct emotions and a random-effects regressors of subjects. The dependent variable was memory performance (remembered – not remembered items). We obtained following exponential Bs (odds ratios): for arousal = 1.15, disgust = 1.72, sadness = 1.3.8 and fear = 1.33 (effect of valence was not significant in the model). Also adding emotional category variables increased amount of variance explained (Negelkerke R Square from 0.033 to 0.04 – low numbers may be connected to very high amount of cases in the model). So the effect of basic emotions (especially the disgust) was stronger than effect of arousal.

### fMRI Results

#### Whole Brain Analysis

**FIGURE 5:**
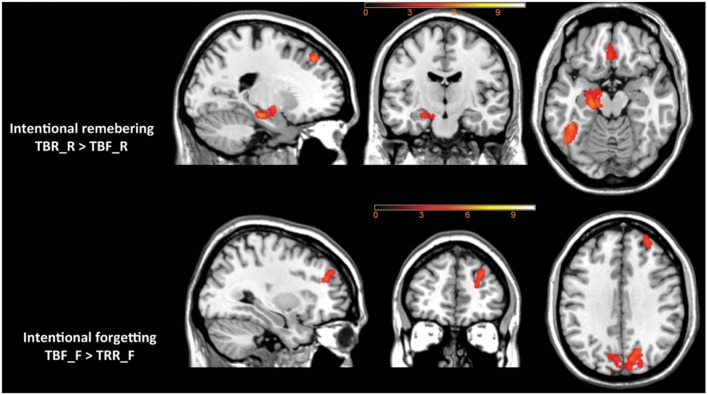
**The effect of intentional remembering and intentional forgetting in the whole brain analysis**.

Intentional remembering (TBR_R > TBF_R) led to activation in medial temporal lobe (MTL) of the left hemisphere including hippocampus and amygdala, left thalamus, bilateral middle occipital areas, left temporal-occipital cortex and left superior and medial orbital frontal cortex (see **Table 3** and **Figure 5** upper panel). Whereas intentional forgetting (TBF_F > TBR_F) produced activation in the right frontal (middle and superior frontal gyrus) and bilaterally in the occipital lobe (lingual gyrus and cuneus; see **Table 3** and **Figure 5** lower panel).

**Table 3:** Peak level activations for the intentional remembering and forgetting contrasts at peak level, FWE cluster corrected at *p* < 0.05.

Region	BA	MNI			*z*	Extent (voxels)
				
		*x*	*y*	*z*		
**Intentional remembering**						
L inferior and middle temporal gyrus, L fusiform gyrus	37	-48	-54	-6	5.03	1581
		-42	-44	-16	4.52	
		-48	-59	-16	4.18	
L thalamus		-2	-24	12	4.88	409
		-6	-30	5	4.27	
L hippocampus, L amygdala, L putamen		-21	-20	-18	4.54	991
		-20	-8	-7	4.52	
		-30	1	-9	4.45	
L superior frontal gyrus		-18	37	45	4.51	326
R middle occipital gyrus		26	-48	27	4.34	378
		24	-32	20	3.78	
L gyrus rectus, L medial orbital gyrus	10	2	34	-19	4.11	348
	11	-3	57	-4	3.73	
		-2	30	-13	3.69	
L middle occipital gyrus, L angular gyrus		-45	-74	26	4.06	466
		-38	-75	35	3.51	
L precuneus, L Verimis_3		-3	-47	12	3.95	404
		0	-42	-10	3.71	
		-6	-54	24	3.64	
**Intentional forgetting**						
L Cerebellum VI, L & R fusiform gyri, L & R lingual gyri		-8	-72	-12	5.75	2195
		20	-53	-12	4.51	
		-3	-65	-6	4.40	
L & R cuneus, L calcarine, L superior occipital gyrus	18	-9	-87	15	4.69	4313
		15	-84	18	4.29	
		20	-69	38	4.06	
R middle and superior frontal gyrus,	10	28	42	26	4.01	736
	9	26	52	35	3.86	
		23	52	20	3.62	
R superior frontal gyrus		17	-3	59	3.85	382
		18	9	62	3.64	
		26	1	59	3.49	


#### ROI Analysis

**FIGURE 6:**
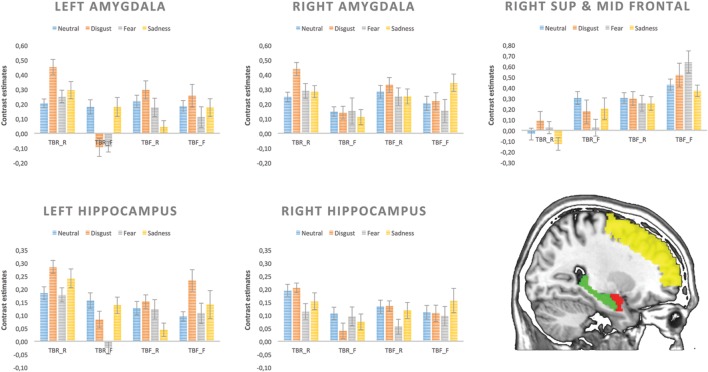
**Contrast estimates extracted for each condition from five regions of interest**.

Contrast estimates from each condition for five ROIs are depicted in **Figure 6.**

As far as the left amygdala is concerned, significant main effects of memory [*F*(1,17) = 7.88, *p* = 0.012] was found, as well as an interaction between instruction and memory outcome [*F*(1,17) = 20.70, *p* < 0.001]. Remembered images resulted in higher left amygdala activity than forgotten ones but this effect was significant only for pictures associated with TBR instruction (TBR_R vs. TBR_F; *p* < 0.001), but not TBF instruction (i.e., TBF_R vs. TBF_F). Additionally, higher left amygdala activity was found for intentional remembering (TBR_R > TBF_R: *p* = 0.001) and intentional forgetting (TBF_F > TBR_F: *p* = 0.023). There was a trend for interaction between emotion and memory outcome [*F*(3,15) = 2.91, *p* = 0.056]. The effect of emotion approached significance only for the remembered stimuli (*p* = 0.061), but not for the forgotten ones. Activity for remembered disgusting images was higher than for all other kinds of images (neutral – *p* = 0.023; fear – *p* = 0.034; sadness – *p* = 0.005). Interestingly, the increased activity for remembered vs. forgotten images was significant only for disgust (*p* = 0.007) and fear – evoking images (*p* = 0.045). In the case of right amygdala, a significant main effect of memory [*F*(1,17) = 9.45, *p* = 0.007] was found and a trend for interaction between instruction and memory outcome [*F*(1,17) = 3.95, *p* = 0.063]. Remembered images resulted in overall higher activity than forgotten ones, but this effect was significant only in case of pictures associated with TBR instruction (*p* = 0.001).

For both hippocampi, a significant interaction of instruction and memory outcome was found [left – *F*(1,17) = 19.55, *p* < 0.001 and right – *F*(1,17) = 6.49, *p* = 0.021]. The remembered images produced higher levels of activity than the forgotten ones but only when they were instructed as to-be-remember (TBR_R vs. TBR_F: left – *p* = 0.001, right – *p* = 0.021), whereas there was no difference for those followed by the TBF instruction (i.e., TBF_R vs. TBF_F). Additionally, higher hippocampi activity was found for intentional remembering (TBR_R vs. TBF_R: left – *p* = 0.001, right – *p* = 0.024).

Last but not least, there were significant main effects of instruction [*F*(1,17) = 9.36, *p* = 0.007] and memory outcome [*F*(1,17) = 9.37, *p* = 0.007] in the right superior and middle frontal gyrus. This time, the activity was higher for the TBF than TBR instruction. Additionally, forgotten images produced more activity than the remembered ones. There were no other significant effects in the right superior and middle frontal gyrus.

## Discussion

The main goal of the current study was to investigate behavioral and neural mechanisms of intentional remembering and forgetting of two types of stimuli: emotionally charged as classified according to the categorical model of affect, and neutral material. To the best of our knowledge, this is the first study where pictures inducing fear, sadness, and disgust were used in an item-method directing forgetting paradigm. The stimuli were selected from the NAPS (Marchewka et al., 2014b) based on the normative ratings collected in line with the basic emotion approach (Riegel et al., 2016b) and a preliminary rating study. In addition, pictures were rated by subjects from the present study, which confirmed the stimuli could induce the intended emotions.

At the behavioral level, recognition rates for to-be-remembered (TBR) and to-be-forgotten (TBF) trials as well as false alarms were analyzed for each of the three emotion categories (disgust, fear, sadness) and neutral images. As expected, we found a significant DF effect, i.e., the recognition rate for TBR was higher than for TBF items and this effect was found for each type of the studied emotions. Intentionally remembered (TBR_R) items were characterized by both better discrimination (Pr) and bias to respond “old” (Br) than incidentally remembered (TBF_R) items, in line with the DF effect. Moreover, similarly to previous studies, the recognition rate was higher for emotional when compared to neutral stimuli (Joormann et al., 2005; Barnier et al., 2007; Nowicka et al., 2011) and this effect was observed for both intentionally and incidentally remembered items. With regard to the basic emotion effects, pictures evoking disgust were better remembered than stimuli evoking sadness and there was a trend in the same direction for images evoking fear. Moreover, pictures evoking disgust had better discrimination and bias to respond “old” than neutral pictures. The same bias was seen in fearful images. Sadness inducing images had better discrimination than both neutral and fearful images, however, they were characterized by a bias to respond “new.” Importantly, analysis based on false alarm corrected recognition revealed significant DF effects (TBR – TBF) effects for all emotions and no interaction between emotion type and type of stimulus. Therefore, it seems that basic emotions have no effect on DF using false alarms corrected recognition. The negative findings obtained in this analysis could be due to the high level of false recognition for stimuli eliciting disgust, since in the typical recognition analysis (not corrected for false alarms), the interaction between basic emotions and stimulus type was significant. Future studies are needed to test whether stimulus driven basic emotions differentially influence false recognition. Additionally, even though subjects’ subjective ratings indicated that disgusting pictures were judged as more arousing and negative than other picture categories, logistic regression on the item level showed that the effect of disgust on recognition memory was stronger than the effect of arousal or valence.

In summary current results could be interpreted as another evidence for a specific role of disgust in memory enhancement beyond those of other negative emotions (Charash and McKay, 2002; Croucher et al., 2011; Chapman et al., 2012). Better memory recognition for disgusting than fearful or sad stimuli, even when specifically asked to forget, might be associated with different influences on attention that these emotions impose. It has been shown that attention disengagement is more difficult from disgust-related than from fear-related stimuli (Cisler et al., 2009; van Hooff et al., 2013, 2014), while sad stimuli probably reduce general alertness by directing attention inward (Finucane et al., 2010). Disgust enhancement of memory can be also linked to the unique contaminating property of disgusting stimuli. Contamination is an effective psychological force since it spreads easily and invisibly between objects (Rozin and Fallon, 1987). Therefore, disgusting and contaminated objects are the ones to be remembered more effectively. In addition, retention of disgust could be especially accurate and enduring because there is a robust correspondence between disgust and conditioned taste aversion (CTA), a particularly powerful form of memory (Garb and Stunkard, 1974; Welzl et al., 2001).

On the neuronal level, we found a typical pattern of activation specific to successful intentional forgetting and intentional remembering consistent with previous studies (Anderson and Hanslmayr, 2014). When compared with incidental memory effects, intentional forgetting engaged prefrontal and occipital areas, suggesting that forgetting is effortful, coherently with behavioral findings. The right superior and middle frontal gyrus (otherwise known as dorsolateral prefrontal cortex, DLPFC) was found to be consistently more active during intentional than incidental forgetting in a number of previous item-method studies (Wylie et al., 2008; Nowicka et al., 2011; Rizio and Dennis, 2012). On the other hand, intentional remembering engaged a left lateralized network of MTL including hippocampus and amygdala and cortical regions in the temporal, parietal, and frontal lobes. The engagement of MTL has been repeatedly shown for successful encoding (Kensinger et al., 2003). Interestingly, connectivity analyses have revealed that activity in the right DLPFC is negatively related to the activity in the MTL and exerts inhibitory control over the MTL and other structures involved during the encoding activity (Paller and Wagner, 2002). Left angular gyrus activity in the intentional remembering is in line with previous studies implicating inferior parietal cortex as belonging to the attention network important for successful encoding of information (Wylie et al., 2008). Bergström et al. (2007) showed in the think/no think paradigm that a parietal positivity was attenuated for learned no-think trials in comparison to learned think trials, which was later confirmed by Mecklinger et al. (2009). Activation in the ventral occipital cortex (including fusiform) was found for both intentional remembering (left lateralized) and intentional forgetting (bilateral). This brain region was implicated in processing of visual details at encoding phase. For instance Kensinger et al. (2007) showed greater activity in the right fusiform, both in extent and in magnitude, during the encoding of negative items due to enhanced visual processing of those stimuli, which resulted in accentuated memory for details. In a previous DF study (Nowicka et al., 2011) this structure was also activated during intentional forgetting, encouraging a hypothesis that precise knowledge of details is also important to efficiently disregard an object when instructed to forget.

To further explore the effect of basic emotions on brain activation, we performed ROI analyses with anatomically defined ROIs controlling for the effect of valence and arousal. In case of the left amygdala the effect of emotion approached significance, but only in the case of remembered stimuli. Disgust eliciting material produced higher activity than all other kinds of images, both neutral and emotional (fearful, sad). The activity was also higher for remembered in comparison to forgotten images but only for those eliciting disgust and fear. In contrast, the activity of right amygdala was not modulated by basic emotions, but only by memory effect – remembered images had increased activation compared to forgotten ones. There might be several explanations as to why we found modulatory effect of emotion only in the left, but not right amygdala. Evidence for a lateralized pattern of amygdala contribution to emotional memory comes from a study on patients with unilateral amygdala lesions. While left amygdala damaged patients failed to show enhanced memory for emotional material both verbal and non-verbal, right amygdala damaged patients showed normal patter of emotional memory facilitation (Buchanan et al., 2001). Another explanation might be related to the sex of the studied sample restricted only to women. FMRI studies suggest that the left and right amygdala could be differentially involved in memory for emotional material depending on the sex of the subjects. Activity in the left amygdala was found to be correlated with memory of emotional stimuli only in female subjects, whereas, in men such pattern was observed in the right amygdala (Canli et al., 2002; Cahill et al., 2004).

Our results are in line with the notion that amygdala activity at encoding influences the likelihood that an emotional item, but not a neutral one, is later remembered (Dolcos et al., 2005; Kensinger and Schacter, 2006). However, in contrast to Kensinger and Schacter (2006) study, where the relationship of the amygdala to subsequent memory was found to be equally strong for all highly arousing positive and negative stimuli, we find this effect only in case of disgusting and fearful images. For sad and neutral images no significant enhancement of amygdala activity was observed for remembered items. One could speculate that such pictures are characterized by lower arousal and thus do not engage amygdala in the same way as highly arousing stimuli. This is, however, unlikely, as arousal and valence were controlled for and their influence was regressed out from the analyses. Moreover, we found that disgusting images engaged amygdala to a larger extent than sad or even fearful pictures. Disgust-evoking stimuli were observed to engage more attentional resources than the fear-relevant cues during the early perceptual processing stage, as indexed by the Early Posterior Negativity (EPN; Wheaton et al., 2013). Furthermore, van Hooff et al. (2014) demonstrated the disgust-specific influence on the very early sensory processing stages in the covert orienting paradigm. These findings suggest that when compared to fearful stimuli disgust-evoking images may require more attentional resources in order to fully assess the potential risk, which is reflected in greater recruitment of the amygdala.

As mentioned above, hippocampal activity was modulated by both memory outcome and instruction, with greater activity for remembered than forgotten stimuli but only those associated with instruction to remember. An opposite effect was seen in the right DLPFC where the activity was higher for the forgotten images than the remembered ones. Additionally, instruction to forget was also associated with higher level of a DLPFC and lower hippocampal activity than the instruction to remember. The activity in these structures, however, was not modulated by emotions in agreement with previous studies showing that hippocampus is involved in the contextual encoding of emotional as well as neutral stimuli (Kensinger and Schacter, 2006).

Altogether, our results suggest the influence of visual complex stimuli eliciting various basic emotions on the memory processes which cannot be simply put down to differences in emotional dimensions. They also indicate that disgusting pictures are distinctive and trigger effects dependent not only on valence and arousal. Instead, it could be argued that disgust has a very specific function, or that disgust-evoking pictures had higher impact, explained as “the immediate effects of images on viewers in terms of their generic cognitive-affective qualities” (Murphy et al., 2010).

## Conclusion

Described here item-method DF fMRI study reveal that memory of emotional information may depend on basic emotional category of stimuli. The disgusting stimuli were found to be the hardest to forget and induced the highest level of left amygdala activity.

One of the limitations of the current experiment is that subgroups of images eliciting basic emotions differed significantly in ratings of valence and arousal. Therefore, we applied statistical tools both on behavioral and neuroimaging level to control for these differences. Consequently behavioral results should be treated with caution despite being in line with previous studies (Chapman et al., 2012) where disgusting and fearful photographs were of similar valence and arousal. Additional research should be also done using verbal material (words) in order to confirm present findings. Another limitation of the experiment is that the study group was limited only to woman and therefore additional work in a group of men should be conducted.

## Author Contributions

Substantial contributions to the: conception or design of the work: AM, JM, KJ, AN; acquisition: AM, MS, MWo, MWy, JM, KJ; analysis: AM, KJ, MWy, JM, MS; interpretation of data for the work: AM, JM, MWy, KJ, AN; writing manuscript: AM, JM, AN, KJ; Final approval of the version to be published: AM, JM, MS, MWo, MWy, KJ, AN. Agreement to be accountable for all aspects of the work in ensuring that questions related to the accuracy or integrity of any part of the work are appropriately investigated and resolved: AM, JM, MS, MWo, MWy, KJ, AN.

## Conflict of Interest Statement

The authors declare that the research was conducted in the absence of any commercial or financial relationships that could be construed as a potential conflict of interest.

## Footnotes

1 https://www.flickr.com/creativecommons/.

2 http://www.alivelearn.net/xjview.

3 http://fmri.wfubmc.edu/software/pickatlas
